# Chitosan Derivatives Associated with Ceftazidime: Study of Controlled Release and Antibacterial Activity

**DOI:** 10.1021/acsomega.5c06901

**Published:** 2025-09-25

**Authors:** Luizângela da Silva Reis, Roosevelt D. S. Bezerra, Humberto M. Barreto, Josy A. Osajima, Edson C. Silva-Filho

**Affiliations:** † Laboratory of Biology, Department of Biology, 67823 Federal University of Piaui (UFPI), Floriano, Piauí 64800-000, Brazil; ‡ Interdisciplinary Advanced Materials Laboratory (LIMAV), Materials Science and Engineering Graduate Program, 67823 Federal University of Piaui (UFPI), Teresina, Piauí 64049-550, Brazil; § Federal Institute of Education, Science and Technology of Piauí (IFPI), Teresina-Central Campus, Teresina, Piauí 64000-040, Brazil; ∥ Microbiology Research Laboratory, 529937 Federal University of Piauí, Ministro Petrônio Portella University Campus, Teresina 64049-550, Piauí Brazil

## Abstract

The search for new low-cost and nontoxic materials with the potential for controlled drug release and antibacterial activity has intensified recently. Due to their unique properties, chitosan derivatives have emerged as promising candidates for these applications. This study evaluated the ability of three chitosan derivatives to incorporate and control the release of the ceftazidime (CFZ) drug, as well as to investigate the antibacterial activity of these derivatives when combined with CFZ against *Staphylococcus aureus* and *Escherichia coli*. Initially, the incorporation of the CFZ drug into chitosan derivatives obtained via sequential modification with acetylacetone (CS-AC) and subsequently with ethylenediamine (CS-AC-EN) or diethylenetriamine (CS-AC-DIEN) was evaluated. The adsorption isotherms were best described by the Temkin model. The maximum adsorption capacities were 24.00 ± 0.20 μg mg^–^^1^ (CS-AC), 20.10 ± 0.70 μg mg^–^^1^ (CS-AC-EN), and 25.50 ± 0.50 μg mg^–^^1^ (CS-AC-DIEN). In gastric medium, the chitosan derivatives exhibited rapid CFZ release within the first 30 min: 47.06 ± 0.80% (CS-AC), 53.84 ± 0.30% (CS-AC-EN), and 21.87 ± 0.60% (CS-AC-DIEN). By contrast, at intestinal pH, the release was slower and controlled, reaching 50.38 ± 0.10% for CS-AC at 72 h, 11.88 ± 0.50% for CS-AC-EN at 24 h, and 5.08 ± 0.30% for CS-AC-DIEN at 6 h. The release profiles were best fitted by the Korsmeyer–Peppas kinetic model. Antibacterial assays against *S. aureus* and *E. coli* showed that all three chitosan derivatives combined with CFZ outperformed CFZ alone and the mixture of unmodified chitosan with CFZ. The advantage was most pronounced after 72 h: against *E. coli*, all derivatives achieved >90% inhibition. These findings indicate that the chitosan derivatives are promising materials for controlling CFZ release in gastric and intestinal environments. Furthermore, when combined with CFZ, these derivatives demonstrate significant potential for antibacterial applications against Gram-positive and Gram-negative bacteria.

## Graphical abstract

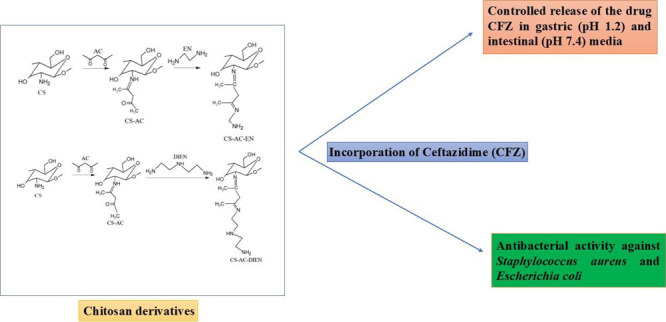

## Introduction

In recent years, increasing resistance to traditional antibiotics has become a growing public health concern due to the emergence of multidrug-resistant bacteria. Moreover, it is estimated that antimicrobial resistance currently causes approximately 1.3 million deaths worldwide, with projections suggesting this number could reach 10 million annually by 2050. It has driven the search for innovative strategies to develop effective tools that enhance the efficacy of existing antibacterial drugs.^1,2^

Thus, Ceftazidime (CFZ) is a hydrophilic antibiotic from the cephalosporin class with a broad-spectrum activity against Gram-positive and Gram-negative bacteria. Its antimicrobial action occurs through binding to penicillin-binding proteins in the bacterial cell wall, preventing the formation of new cell structures and inhibiting bacterial growth. CFZ also exhibits resistance to various β-lactamases, a medication indicated for biliary tract infections, bone and joint infections, respiratory tract infections, and urinary tract infections.^3–5^

In this context, the search for new compounds with antimicrobial potential, low-cost, and the ability to facilitate the incorporation and controlled release of proven agents has garnered significant interest within the scientific community. Chitosan (CS) and its derivatives have been widely used in controlled drug release systems and antimicrobial activity studies against various pathogens.^6–12^

Chitosan (CS) is the second most abundant biopolymer in nature and stands out for its attractive characteristics, including low-cost, nontoxicity, biodegradability, and excellent biocompatibility. Additionally, chitosan (CS) contains amino (NH_2_) and hydroxyl (OH) functional groups, which can be readily modified chemically, enhancing the versatility of this biopolymer. The incorporation of new functional groups into its structure not only improves its biological properties but also significantly expands the potential applications of its derivatives.^13,14^

For example, a study demonstrated that the chemical modification of chitosan (CS) significantly enhanced its efficacy against *Staphylococcus aureus* and *Escherichia coli*. The modified material (m-Ch) exhibited an inhibition rate of 98.2 ± 0.84% against *S. aureus* and 24.85 ± 6.51% against *E. coli*, while unmodified chitosan (Ch) showed only 58.11 ± 1.46% inhibition for *S. aureus*. These findings suggest that chitosan functionalization can enhance its antimicrobial activity, particularly against Gram-positive bacteria.^15^ Another study revealed that Schiff bases derived from chitosan exhibited higher antimicrobial activity than Amoxicillin and Tetracycline. Additionally, these bases showed no cytotoxicity compared to Colchicine.^16^

Furthermore, chitosan and its derivatives are highly promising materials for drug delivery, including anticancer agents, antibiotics, anti-inflammatory drugs, and proteins.^17^ These materials provide advantageous properties for controlled drug delivery systems, such as thermal responsiveness and pH dependency. The thermoresponsive and pH-sensitive nature of the polymer allows for more precise drug release control, enabling more efficient and targeted administration.^18^

In this regard, further studies are essential to enhance the properties of chitosan, both in terms of controlled drug release and its antibacterial activity against a wide range of pathogens. It is crucial to explore various chemical modifications to increase its effectiveness and expand its biomedical applications. In this context, a study is needed on the modification of chitosan (CS) with acetylacetone and, subsequently, with ethylenediamine and diethylenetriamine, and the application of these chitosan derivatives in the incorporation/controlled release of the drug ceftazidime (CFZ) and studies of antibacterial activity against Gram-positive and Gram-negative bacteria, considering that there are no reports in the literature for this type of study.

Therefore, the present study aims to investigate the incorporation and controlled release of the drug ceftazidime (CFZ) in chitosan derivatives (CS) and evaluate the inhibitory effect of these modified materials incorporated with the drug against the bacteria *S. aureus* and *E. coli*.

## Materials and Methods

### Materials

Chitosan (CS) (medium degree of deacetylation 78% and molar mass 132.0 kDa),^7,8^ sodium hydroxide (DINÂMICA), acetylacetone (Sigma-Aldrich), ethylenediamine (Sigma-Aldrich), diethylenetriamine (Sigma-Aldrich), ceftazidime pentahydrate (CFZ) (BIO CHIMICO), acetic acid (VETEC), Brain Heart Infusion (BHI) (HIMEDIA), Nutrient Agar (HIMEDIA), GM07492A (Human Fibroblast), DMEM (Gibco/Thermofisher), supplemented with Fetal Bovine Serum (FBS) (Nutricell), penicillin, streptomycin 10 U mL^–1^ (Sigma-Aldrich) and sodium chloride (IMPEX). The reagents were all of analytical grade and used without prior purification.

### Chemical Modifications of Chitosan (CS)

The modification of Chitosan (CS) with acetylacetone (AC) and, subsequently, with ethylenediamine (EN) and diethylenetriamine (DIEN) was performed as shown in Figure 1, according to an article published by our research group in the work of Pereira et al. (2019).^7^

**Figure 1:**
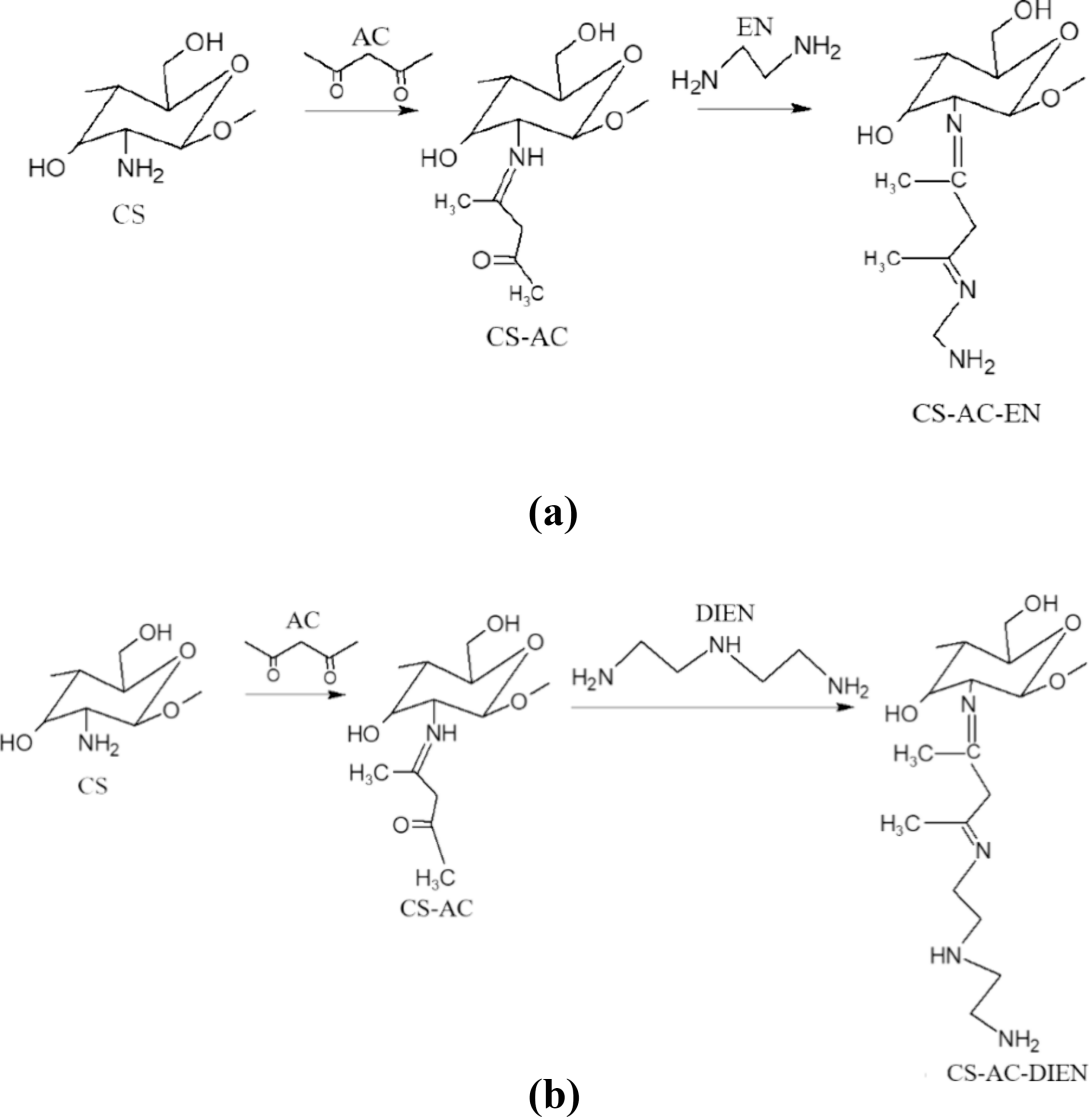
Proposed reaction scheme for the chemical modification of chitosan (CS) with acetylacetone (AC) and, subsequently, with ethylenediamine (EN) (a) and diethylenetriamine (DIEN) (b).

### Characterization of Chitosan (CS) and Its Derivatives

Chitosan and its derivatives were characterized by elemental analysis (CHN), infrared spectroscopy (FTIR), X-ray diffraction (XRD), ^13^C nuclear magnetic resonance (^13^C NMR), and thermogravimetric analysis (TGA-DTG-DSC). The results of these characterizations have already been published by our research group in the work of Pereira et al.^7^

### Incorporation of Ceftazidime (CFZ) in the Chitosan Derivatives

The adsorption isotherms for the incorporation of the drug ceftazidime (CFZ) in the chitosan derivatives (CS-AC, CS-AC-EN, and CS-AC-DIEN) were performed in triplicates and at a temperature of 298 K. In this study, a stock solution of CFZ was prepared, and then several solutions were prepared with concentrations ranging from 50 to 225 μg mL^–^^1^ of the respective drug. Subsequently, 20.0 mL aliquots of each solution were placed in Erlenmeyer flasks containing approximately 40.0 mg of each sample (in powder). Immediately after, the samples were shaken for 48 h at 130 rpm on a shaker table. After this agitation period, the supernatant was separated by centrifugation, and the final concentrations of the samples were determined using UV–vis Spectroscopy (Varian CARY 60 model) at a wavelength of λ_max_ = 257 nm. The amount of adsorbed drug (*q*_e_) (μg mg^–^^1^) by the materials was calculated according to eq 1.^6,19,20^

$$q_{e}=\frac{\left(C_{i}-C_{f}\right)}{m}.V$$ 1

Where *q*_e_ (μg mg^–^^1^) indicates the amount of drug adsorbed per unit mass of the adsorbent, *C*_i_ and *C*_f_ (μg mL^–^^1^) correspond to the initial and final concentrations of the drug in the solution, respectively, *V* (L) represents the volume of the solution, and *m* (g) is the mass of the adsorbent.

The experimental data of the adsorption isotherms were fitted to the Langmuir,^21^ Freundlich,^22^ and Temkin^23^ models, as shown in eq 2, eq 3, and eq 4, respectively.

$$\frac{C_{e}}{q_{e}}=\frac{C_{e}}{q_{\max}}+\frac{1}{K_{L}q_{\max}}$$ 2

$$\ln\;q_{e}=\ln\;K_{F}+\frac{1}{n}\ln\;C_{e}$$ 3

$$q_{e}=\frac{1}{n_{T}}\ln\;K_{T}+\frac{1}{n_{T}}\ln\;C_{e}$$ 4

In these equations, *q*_e_ (mg g^–1^) represents the amount of substance adsorbed per unit mass of the adsorbent, while *q*_max_ (mg g^–1^) corresponds to the maximum adsorption capacity of the adsorbent. The equilibrium concentration of the adsorbate is denoted by *C*_e_ (mg L^–1^), whereas *K*_L_ represents the Langmuir adsorption constant associated with the chemical equilibrium between the adsorbate and the adsorbent. Meanwhile, *K*_F_ for the Freundlich adsorption constant and *n* is a parameter that reflects the strength of the adsorption process. n_T_ indicates the quantitative reactivity of the energetic sites, while *K*_T_ is a constant that encompasses the equilibrium constant.^24–26^

### In Vitro Drug Release Study

The simulated fluids were prepared according to protocols already described in the literature.^27–29^ For simulated gastric fluid (SGF), 2.0 g of NaCl was dissolved in sufficient water for complete solubilization. Then, 7.0 mL of concentrated HCl was added and the volume was made up to 1.0 L with Milli-Q water. During the addition of the acid, the pH was monitored and maintained at 1.2 ± 0.1. Simulated intestinal fluid (SIF) was prepared by dissolving 21.7 g of dibasic sodium phosphate and 2.6 g of monobasic potassium phosphate in 1 L of deionized water. The pH was then adjusted to 6.8 or 7.4 with 0.1 mol L^–^^1^ NaOH or HCl solution.

### Drug Release Study

The study of controlled release of the drug CFZ by the materials (CS-AC, CS-AC-EN, and CS-AC-DIEN) was conducted by simulating gastric fluid (pH 1.2) and intestinal fluid (pH 7.4), according to the methodology proposed by Silva et al. (2020).^27^ In this study, a 20.0 mg sample (in powder) was placed in a dissolution medium containing 50 mL of a pH-controlled solution and subjected to a water bath with agitation at 100 rpm, maintaining a temperature of 310 ± 5 K. Subsequently, at 1, 2, 4, 5, 24, 48, 72, 96, and 120 h, 3.0 mL aliquots of the release medium were withdrawn and immediately replaced with the same volume of solution in order to maintain a constant total volume, and the CFZ concentrations were quantified using UV/vis spectrophotometry at 257 nm. All analyses were performed in triplicate.

Furthermore, the experimental data from the controlled release study of the CFZ drug were fitted to the kinetic release models described in eqs 5, 6, 7, 8, and 9.^27,30–32^

Zero-order model:

$$q_{t}=q_{0}+K_{0}t$$ 5

First-order model:

$$\ln C={\ln C}_{0}-K_{1}t$$ 6

Korsmeyer-Peppas model:

$$\frac{M_{t}}{M_{\infty }}=Kt^{n}$$ 7

Higuchi model:

$$F=K_{2}t^{0.5}$$ 8

Hixson-Crowell model:

$$W_{t}^{1/3}=W_{0}^{1/3}-K_{3}t$$ 9

In these equations, *K*_0_, *K*_1_, *K*, *K*_2_, and *K*_3_ are the release constants for each model. Meanwhile, *q_t_*, *C*, *M_t_*, *F*, and *W_t_* are related to the amount of drug released at time *t*. Finally, *q*_0_, *C*_0_, and *W*_0_ correspond to the initial amounts of the drug, while *M*_∞_ represents the amount of drug released at infinite time. The value of n characterizes the drug release mechanism.

### Evaluation of Antibacterial Activity

The assays for evaluating antibacterial activity were conducted using a Gram-positive strain ( *S. aureus* ) (ATCC 25923) and a Gram-negative strain (*E. coli*) (ATCC 25922). All strains were maintained on nutrient agar at 4 °C. The bacterial cultures were prepared following methodologies previously described in the literature.^6,8,13,14,33^ At the end of the process, suspensions of approximately 1.5 × 10^4^ CFU mL^–1^ for *S. aureus* and 1.5 × 10^5^ CFU mL^–1^ for *E. coli* were obtained.

This study prepared solutions of CFZ (1000 μg mL^–1^) and chitosan derivatives (1000 μg mL^–1^) using 2% acetic acid as the solvent. Thus, 100 μL of the standardized suspension was transferred to Petri dishes containing nutrient agar. Then, 100 μL of the test solution was added to each plate. The inoculation was performed using the spread plate method with the aid of a Drigalski spatula, and the plates were incubated at 310 K for 24 h. As a positive growth control, nutrient agar plates containing the bacterial suspension and saline solution were used, as well as plates containing the bacterial suspension and 2% acetic acid solution. All assays conducted with the test and control solutions were performed in triplicate. The inhibitory effect produced by each test solution was calculated using the following eq 10:

$$\eta =\frac{N_{1}-N_{2}}{N_{1}}\times 100$$ 10

Where η represents the inhibitory effect, *N*_1_ is the arithmetic mean of the colony-forming units (CFU) in the control plates, and *N*_2_ is the arithmetic mean of the colony-forming units in each tested solution.

### Statistical Analysis

The statistical significance of differences between groups was determined using analysis of variance (ANOVA), followed by Tukey’s posthoc test. Differences were considered statistically significant at *p* < 0.05.^8,34^

## Results and Discussion

### Characterization of Chitosan Derivatives

The chemical modification of chitosan (CS) with acetylacetone (AC), followed by ethylenediamine (EN) and diethylenetriamine (DIEN), as proposed in the mechanism shown in Figure 1, was carried out by our research group and published in the study by Pereira et al.^7^ In this study, our research group confirmed the incorporation of amine groups into the chitosan structure through elemental analysis (CHN), FTIR, XRD, ^13^C NMR, and thermogravimetric analysis (TGA-DTA-DSC) after the chemical reactions shown in Figure 1.

### Incorporation of Ceftazidime (CFZ) in the Chitosan Derivatives

Figure 2 presents the experimental data of the adsorption isotherm for incorporating CFZ by chitosan derivatives (CS-AC, CS-AC-EN, and CS-AC-DIEN). From the curves, it can be observed that as the concentration of the CFZ solution increases, adsorption also rises for the three derivatives, reaching maximum values of 24.00 ± 0.20 μg mg^–^^1^ for CS-AC, 20.10 ± 0.70 μg mg^–^^1^ for CS-AC-EN, and 25.50 ± 0.50 μg mg^–^^1^ for CS-AC-DIEN. These chitosan derivatives exhibited CFZ incorporation values higher than those obtained for pure chitosan. According to literature data published by our research group,^6^ pure chitosan incorporates approximately 8.00 μg mg^–^^1^.

**Figure 2:**
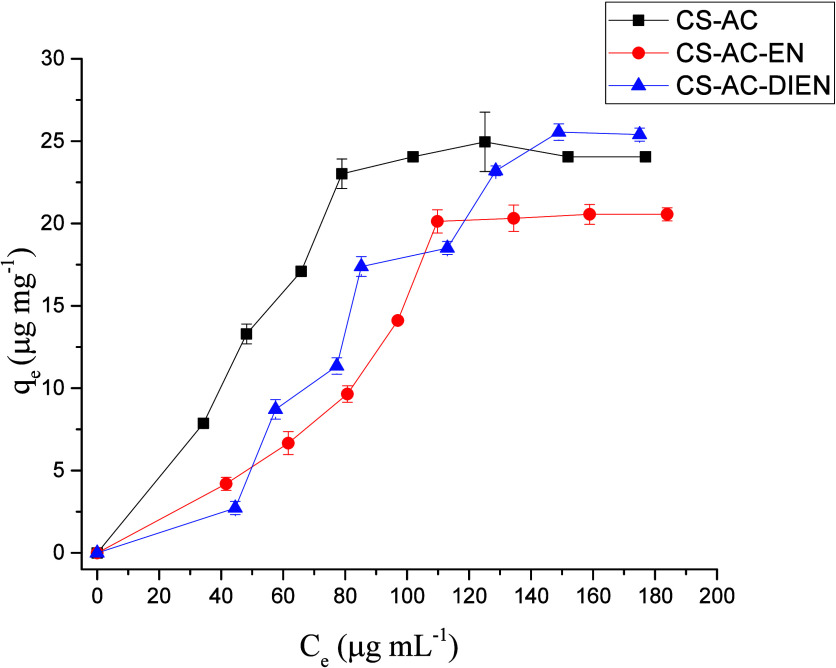
CFZ adsorption isotherms on chitosan derivatives (CS-AC, CS-AC-EN, and CS-AC-DIEN) at 298 K.

This result is important as it demonstrates that the chemical modifications in the chitosan derivatives increased the amount of incorporated CFZ drug. When comparing the amounts of CFZ adsorbed by CS-AC, CS-AC-EN, and CS-AC-DIEN with pure chitosan, an increase in adsorption capacity of 300% for CS-AC, 250% for CS-AC-EN, and 312.5% for CS-AC-DIEN are observed. These results confirm that incorporating functional groups in the chemical modification reactions of chitosan enhanced its interaction with the CFZ drug, leading to a significant increase in adsorption.

Moreover, it is important to highlight that, as previously mentioned, the adsorption of CFZ by the three chitosan derivatives (CS-AC, CS-AC-EN, and CS-AC-DIEN) increases as the CFZ concentration rises, until the adsorption rate becomes constant, characterizing the equilibrium of the adsorption process. This behavior occurs because, initially, free active sites are available on the surface of the adsorbents, which are gradually occupied by CFZ molecules. As these sites become saturated, the proximity between the adsorbed molecules generates electrostatic repulsion, limiting further interactions and leading to the stabilization of the adsorption process.^19,25^

The results shown in Figure 2 indicate that the amounts of CFZ adsorbed by the chitosan derivatives vary significantly from one another, which is directly related to differences in the functional groups present in each material. The CS-AC-DIEN derivative exhibited the highest adsorption capacity, attributed to the greater number of available amino groups, which promote the formation of hydrogen bonds with CFZ. In contrast, the CS-AC-EN derivative displayed the lowest adsorption capacity among the three materials. This inferior performance may be associated with the fact that the conversion of CS-AC to CS-AC-EN eliminates the carbonyl groups present in CS-AC, which act as strong hydrogen bond acceptors. The reduction in the number of exposed carbonyls, combined with the presence of a bulkier substituent, decreases the availability of sites for hydrogen bonding and specific interactions with CFZ. Furthermore, ethylenediamine (EN) introduces only one terminal amine in a short spacer, which may favor the formation of intra/interchain interactions, resulting in a more compact matrix with reduced accessibility to active sites. Conversely, diethylenetriamine (DIEN) has a longer and more flexible spacer with additional amines, increasing the exposure of binding sites and enhancing interactions with CFZ.^19,20,25,35^

The experimental adsorption isotherm data were fitted according to the Langmuir, Freundlich, and Temkin models, with the correlation coefficient (*R* ^2^) values in Table 1. The *R* ^2^ values indicate that, for the three CS derivatives, the Temkin isotherm model provided the best fit to the adsorption data, with correlation coefficients of 0.8196, 0.8704, and 0.9553 for CS-AC, CS-AC-EN, and CS-AC-DIEN, respectively. The Temkin model considers the interactions between the adsorbent and the adsorbate, suggesting that due to these interactions, the adsorption heat of all molecules in the layer decreases linearly as the adsorbent surface becomes covered.^35,36^

**Table 1:** Correlation Coefficient (*R* ^2^) Was Obtained from the Linearized Equations of Langmuir, Freundlich, and Temkin

model	CS-AC	CS-AC-EN	CS-AC-DIEN
*q*_max. Exp._ (μg mg^–1^)	24.00 ± 0.20	20.10 ± 0.70	25.50 ± 0.50
Langmuir			
*R* ^2^	0.7292	0.3373	0.2133
Freundlich			
*R* ^2^	0.7722	0.8331	0.6949
Temkin			
*R* ^2^	0.8196	0.8704	0.9553

### In Vitro Release Study

The studies on CFZ release from chitosan derivatives, conducted at pH 1.2 (simulated gastric fluid) and pH 7.4 (simulated intestinal fluid), are presented in Figure 3. According to Figure 3, at pH 1.2, the CS-AC derivative exhibited a rapid release during the initial measurements, reaching approximately 47.06 ± 0.80% within around 30 min. After this period, the release stabilized, showing no significant variations over time. At pH 7.4, the CS-AC exhibited a slower and sustained release over time, reaching approximately 50.38 ± 0.10% after 72 h.

**Figure 3:**
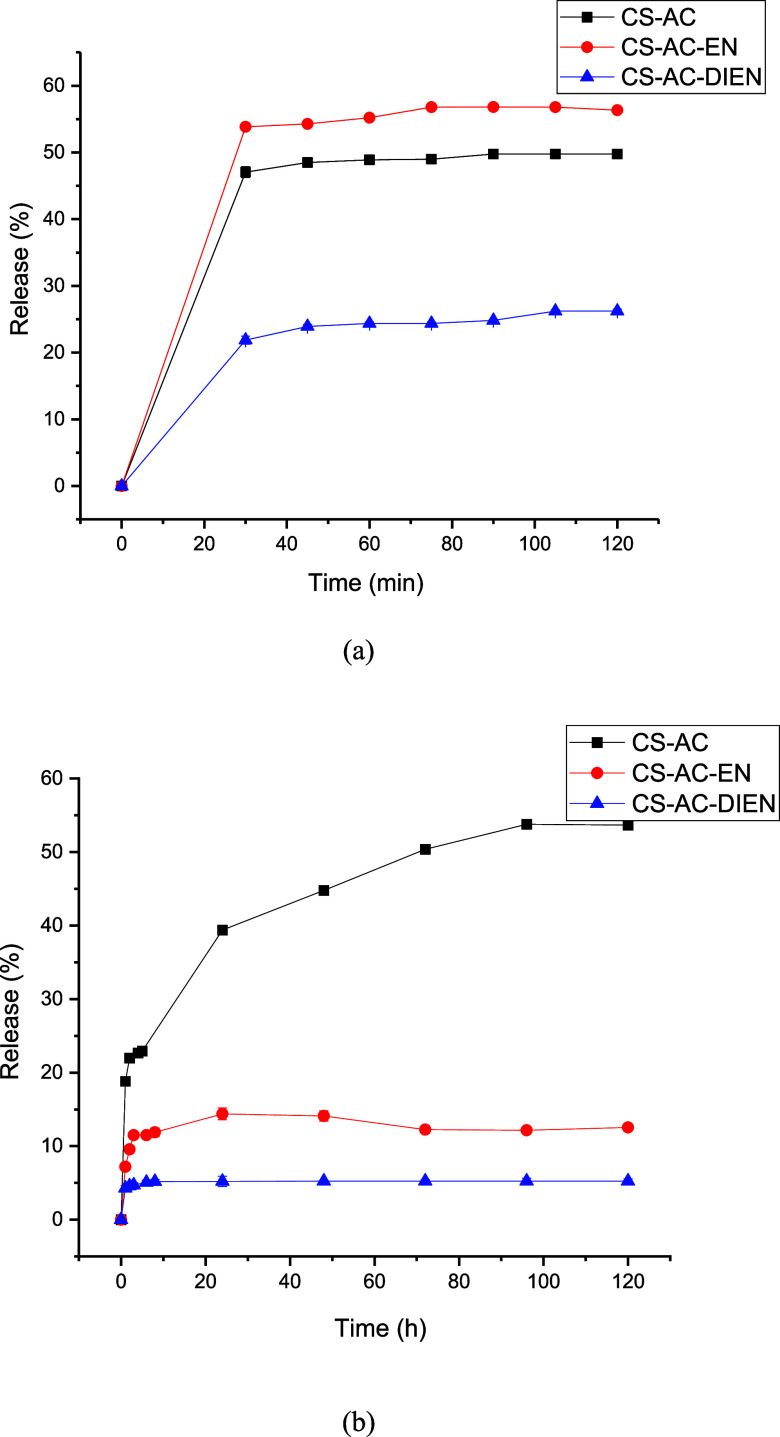
Release profiles of CFZ incorporated in the chitosan derivatives at pH 1.2 (a) and pH 7.4 (b).

The change in the drug release profile of CFZ by the CS-AC derivative during the transition from gastric pH to intestinal pH is directly related to the electrostatic interactions between the drug and the CS-AC material. The CFZ drug acquires a positive charge at gastric pH, while the CS-AC derivative is also protonated. It increases electrostatic repulsion between them, leading to a faster release of the drug from the surface of the CS-AC material. At intestinal pH, the CFZ drug acquires a negative charge, which enhances the electrostatic attraction between it and the CS-AC derivative. Additionally, hydrogen interactions also occur between them, further strengthening this interaction. As a result, the drug release in this medium is slower and more sustained.^37–40^

The drug release profiles of CFZ for the CS-AC-EN and CS-AC-DIEN derivatives were similar to that of the CS-AC derivative. In the gastric medium, the drug was rapidly released within the first 30 min, reaching a maximum release of 53.84 ± 0.30% for CS-AC-EN and 21.87 ± 0.60% for CS-AC-DIEN, with stabilization after this period. In contrast, in the intestinal medium, the release was slower and more controlled, reaching a maximum of 11.88 ± 0.50% after 24 h for CS-AC-EN and 5.08 ± 0.30% after 6 h for CS-AC-DIEN. Another important result is that the amount of drug released by the CS-AC-EN and CS-AC-DIEN derivatives was lower at pH 7.4 than at pH 1.2. This behavior is related to the presence of amino (NH_2_) groups incorporated into these derivatives after the chemical reaction with ethylenediamine (EN) and diethylenetriamine (DIEN). Increasing the number of amino groups enhances electrostatic interactions and hydrogen bonding between the drug and these derivatives, stabilizing CFZ on their surface and making its release more complex.^37–40^

These results confirm that pH variation is crucial in releasing the CFZ drug from the CS-AC, CS-AC-EN, and CS-AC-DIEN chitosan derivatives. The rapid initial release of CFZ can be beneficial for immediately eliminating bacteria and preventing their uncontrolled multiplication. On the other hand, a more controlled release is essential for eradicating remaining organisms after the initial phase, ensuring prolonged and effective therapeutic action. Furthermore, since antibiotics tend to be less stable in acidic environments and may exhibit low penetration through the gastric barrier, using these derivatives can be an effective strategy to enhance the drug’s stability in the stomach and ensure its prolonged release. Thus, these chitosan derivatives are promising materials for sustained drug release in both acidic and alkaline environments, allowing for gradual release in the stomach and the remaining drug to be released in the intestine, optimizing its therapeutic efficacy.^40^

The experimental data on CFZ drug release from chitosan derivatives were fitted to different kinetic models, including zero-order, first-order, Korsmeyer-Peppas, Higuchi, and Hixson-Crowell, to analyze the drug release mechanism of these materials. The correlation coefficient values (R^2^) for each model are presented in Table 2. Based on these results, it can be observed that all CFZ drug release profiles from the CS-AC, CS-AC-EN, and CS-AC-DIEN derivatives best fit the Korsmeyer–Peppas kinetic model, as this model exhibited the highest correlation coefficient (R^2^) values.

**Table 2:** Correlation Coefficient (*R* ^2^) Obtained from the Linearized Equations of Zero-Order, First-Order, Korsmeyer-Peppas, Higuchi, and Hixson-Crowell

				correlation coefficient (*R* ^2^)		
release models		material	pH = 1.2	*n*	pH = 7.4	*n*
zero-Order		CS-AC	0.7829		0.8568	
		CS-AC-EN	0.6795		0.0802	
		CS-AC-DIEN	0.8564		0.3107	
first-Order		CS-AC	0.7768		0.7889	
		CS-AC-EN	0.6816		0.0879	
		CS-AC-DIEN	0.8471		0.2972	
Korsmeyer–Peppas		CS-AC	0.9068	0.0393	0.9755	0.2392
		CS-AC-EN	0.7980	0.0426	0.5109	0.0871
		CS-AC-DIEN	0.9000	0.1198	0.7371	0.0369
Higuchi		CS-AC	0.8533		0.9655	
		CS-AC-EN	0.7403		0.2402	
		CS-AC-DIEN	0.8864		0.4880	
Hixson-Crowell		CS-AC	0.8039		0.8111	
		CS-AC-EN	0.6892		0.0829	
		CS-AC-DIEN	0.8384		0.4013	

The Korsmeyer–Peppas kinetic model exponentially correlates drug release with time and the fraction of drug released.^31^ The *n* value in the Korsmeyer can determine the primary drug release mechanism–Peppas kinetic model (eq 7). The release follows a Fickian diffusion mechanism when *n* ≤ 0.45. For *n* values between 0.45 and 0.89, the transport is classified as anomalous (non-Fickian). If *n* = 0.89, the release follows the case II model, while values greater than 0.89 indicate super case II transport.^38,40^ Table 2 presents the chitosan derivatives’ n values for each CFZ drug release profile. Based on these results, it can be observed that all drug release kinetics exhibited *n* values ≤ 0.45, indicating that the primary release mechanism of CFZ follows a Fickian diffusion pattern. This means that the drug release is purely diffusion-controlled, with the drug diffusing more rapidly through the polymer matrix than the process of polymer chain relaxation.^41^

### Evaluation of Antibacterial Activity

The results of the inhibitory effect of chitosan derivatives associated with the CFZ drug against *S. aureus* and *E. coli* after 48 and 72 h of sample preparation are presented in Figures 4 and 5. Figure 4 shows that the CS-AC-CFZ derivative exhibited the lowest inhibitory effect against the Gram-positive bacterium *S. aureus* , with 40.0 ± 3.0% after 48 h and 65.0 ± 2.0% after 72 h. In contrast, the CS-AC-EN-CFZ and CS-AC-DIEN-CFZ derivatives demonstrated the highest inhibitory effects. The CS-AC-EN derivative reached 95.0 ± 1.0% after 48 h and 97.0 ± 1.0% after 72 h, while the CS-AC-DIEN derivative showed values of 95.0 ± 1.0% after 48 h and 96.0 ± 1.0% after 72 h.

**Figure 4:**
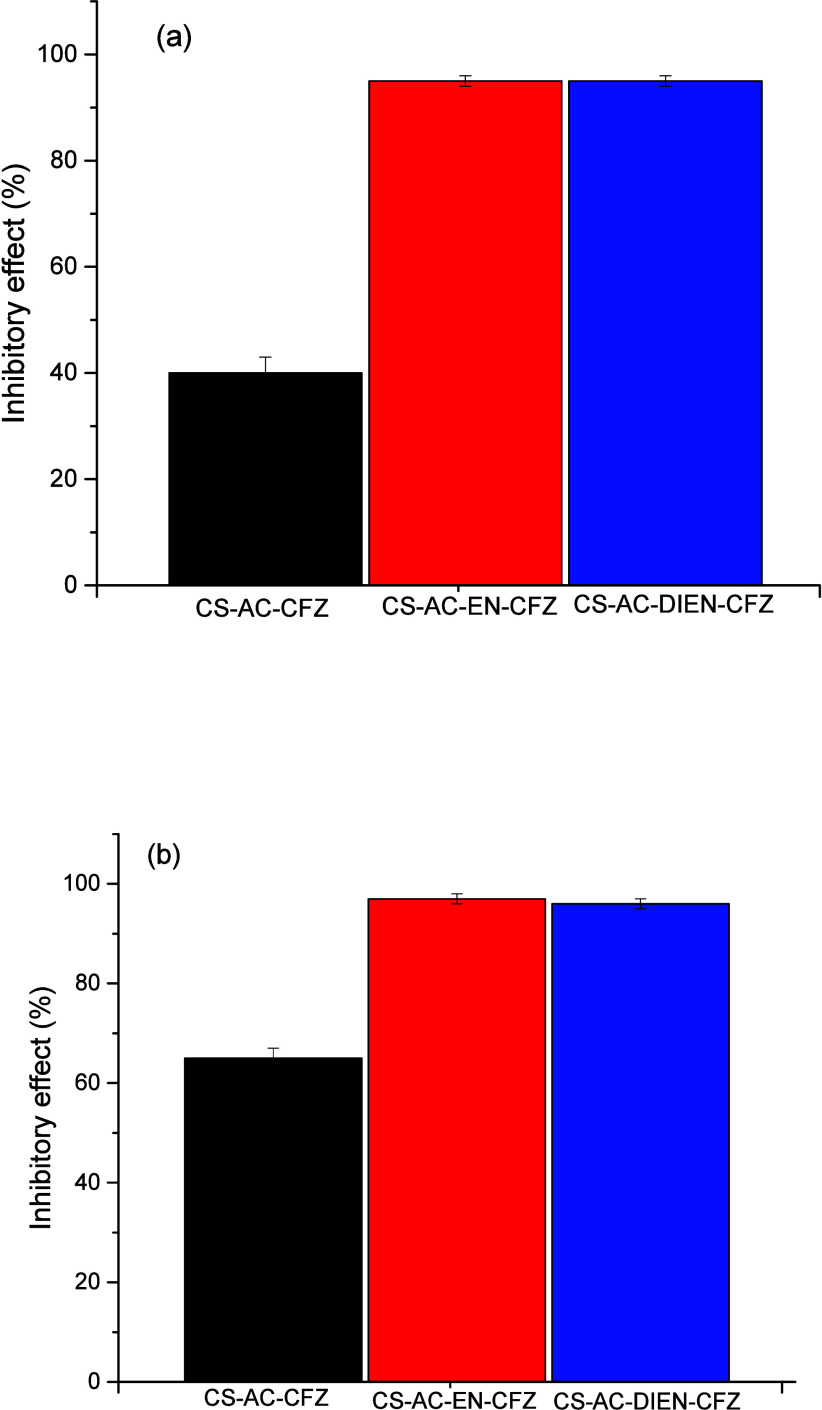
Inhibitory effect of chitosan derivatives associated with CFZ drug against *S. aureus* after (a) 48 h and (b) 72 h of solution preparation (1000 μg mL^–1^, pH = 2.3).

**Figure 5:**
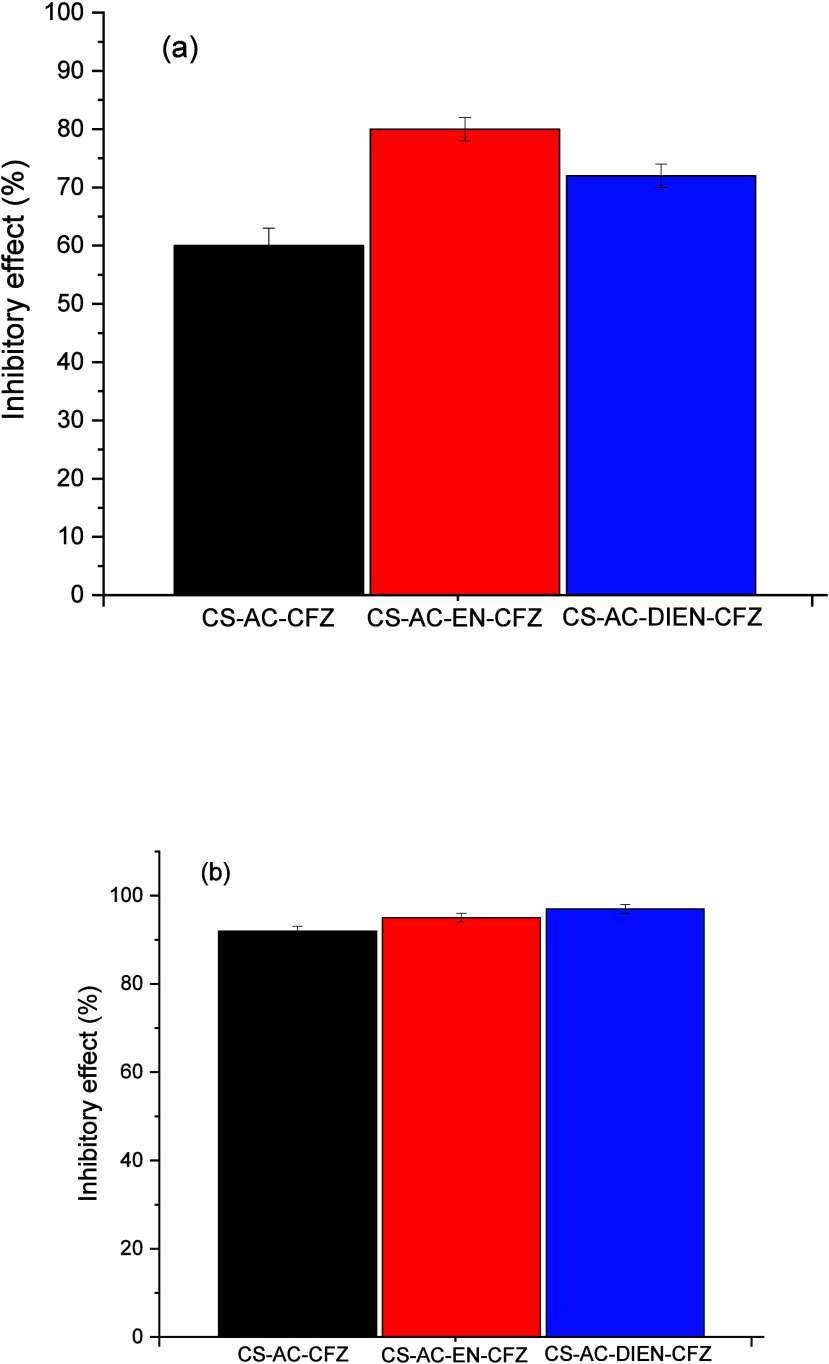
Inhibitory effect of chitosan derivatives associated with CFZ drug against *E. coli* after (a) 48 h and (b) 72 h of solution preparation (1000 μg mL^–1^, pH = 2.3).

The higher inhibitory values of the CS-AC-EN-CFZ and CS-AC-DIEN-CFZ derivatives, compared to CS-AC–CFZ, against *S. aureus* are associated with the incorporation of amino groups after reactions with ethylenediamine and diethylenetriamine. Studies indicate that the presence of amino groups (NH_2_) enhances the inhibitory effect due to the electrostatic interaction between these protonated groups and the negatively charged teichoic and lipoteichoic acids in the bacterial cell wall, leading to increased antibacterial activity.^13,42–44^

Furthermore, the CS-AC-EN-CFZ and CS-AC-DIEN-CFZ derivatives exhibited a higher inhibitory effect against the Gram-positive bacterium *S. aureus* compared to pure chitosan combined with CFZ and pure CFZ alone.^6^ It can be attributed to the synergistic action between the amino groups present in the structure of these chitosan derivatives and the CFZ drug incorporated on the surface of these materials. This interaction enhanced the antibacterial activity, making them more effective against this bacterium.^44,45^

Figure 5 presents the results of the inhibitory effect of chitosan derivatives associated with the CFZ drug against the Gram-negative bacterium *E. coli* after 48 and 72 h. This graph shows that the CS-AC–CFZ derivative exhibits a higher inhibitory effect against the Gram-negative bacterium *E. coli* (60.0 ± 3.0% after 48 h and 92.0 ± 1.0% after 72 h) compared to the Gram-positive bacterium **S. aureus*.*

This result may be associated with the structural differences between the cell walls of Gram-negative and Gram-positive bacteria. In Gram-negative microorganisms, the cellular organization includes two layers external to the cytoplasmic membrane: (i) a thin peptidoglycan layer composed mainly of N-acetylglucosamine and N-acetylmuramic acid cross-linked by short peptides, and (ii) an outer membrane rich in phospholipids and lipopolysaccharides (LPS), the latter containing negatively charged phosphate and carboxyl groups. The acetylacetone (AC) moieties in the CS-AC–CFZ derivative can establish hydrogen bonds and hydrophobic interactions with phospholipid tails, as well as electrostatic interactions between protonated amino groups of chitosan and the anionic sites of LPS. These combined interactions compromise the integrity of the outer membrane, increasing its permeability. This disruption can lead to cytoplasmic condensation and expansion of the intracellular space, ultimately enhancing antibacterial activity against *E. coli*.^13,14,44,45^ Additionally, the synergistic action between the acetylacetone (AC) groups of the CS-AC–CFZ derivative and the CFZ drug incorporated into this material resulted in a more significant inhibitory effect against *E. coli* after 72 h, surpassing both pure chitosan + CFZ and pure CFZ.^6^ These findings indicate that this material is promising for combating Gram-negative bacteria.^44,45^

Finally, the CS-AC-EN-CFZ and CS-AC-DIEN-CFZ derivatives exhibited a lower inhibitory effect against the Gram-negative bacterium *E. coli* after 48 h (80.0 ± 2.0% for CS-AC-EN-CFZ and 72.0 ± 2.0% for CS-AC-DIEN-CFZ) compared to the Gram-positive bacterium *S. aureus* over the same period. This result may be related to the structural differences in the cell wall of Gram-negative bacteria, as discussed earlier.

However, after 72 h, the CS-AC-EN-CFZ and CS-AC-DIEN-CFZ derivatives exhibited inhibitory values of 95.0 ± 1.0 and 97.0 ± 1.0%, respectively, against *E. coli*. These values are statistically similar to those observed against *S. aureus* . They are higher than those reported in the literature for pure chitosan + CFZ and even pure CFZ against *E. coli* within the same period.^6,44,45^ These results indicate that the CS-AC-EN-CFZ and CS-AC-DIEN-CFZ derivatives are promising materials for antibacterial applications against Gram-negative and Gram-positive bacteria.

Based on the results, the envisioned delivery format for ceftazidime (CFZ) using chitosan (CS) derivatives is an oral administration system, such as capsules or tablets. The experiments were conducted in simulated gastric fluid (pH 1.2) and simulated intestinal fluid (pH 7.4). The results showed a markedly different release behavior in these two media. In the acidic environment of the stomach, we observed a rapid initial release of the drug, whereas at the intestinal pH, the release was significantly slower and more controlled. This behavior is ideal for oral administration. A rapid initial release in the stomach can be beneficial for immediate antimicrobial action. Subsequently, the slow and sustained release in the intestine ensures that the drug is released gradually over time, maintaining an effective and prolonged therapeutic concentration.^40^

Among the three derivatives studied, CS-AC-DIEN emerges as the most promising candidate for developing this CFZ delivery system. This is because CS-AC-DIEN showed the highest maximum CFZ adsorption capacity, with a value of 25.50 ± 0.50 μg mg^–^^1^. A higher adsorption capacity means the material is a more efficient carrier, able to transport a larger amount of drug per unit mass. CS-AC-DIEN also demonstrated the most controlled and sustained release profile in intestinal pH (7.4), releasing only 5.08 ± 0.30% of the drug in 6 h. This behavior is ideal for prolonged therapy, as it prevents concentration peaks and maintains the antibiotic’s action for a longer period. Furthermore, CS-AC-DIEN stood out for its high effectiveness against both bacterial strains. After 72 h, it achieved an inhibitory effect of 96.0 ± 1.0% against *S. aureus* (Gram-positive) and 97.0 ± 1.0% against *E. coli* (Gram-negative). This potent, broad-spectrum activity, which is superior to that of pure CFZ and pure chitosan with CFZ, confirms the enormous synergistic potential of this combination.

## Conclusions

This study demonstrated that incorporating the CFZ drug was effective in the CS-AC, CS-AC-EN, and CS-AC-DIEN chitosan derivatives, exhibiting higher amounts of adsorbed CFZ than pure chitosan. Furthermore, the incorporation study revealed that, for all three derivatives, the adsorption isotherms followed the Temkin model. The controlled drug release tests in an aqueous medium demonstrated that these three chitosan derivatives effectively release CFZ in both gastric (pH 1.2) and intestinal (pH 7.4) environments. The release occurs rapidly in the gastric medium, whereas in the intestinal medium, it is more controlled and sustained, ensuring a gradual release of the drug over time. The study of antibacterial activity against *S. aureus* and *E. coli* revealed that these three chitosan derivatives associated with the CFZ drug exhibit a higher inhibitory effect than pure CFZ and pure chitosan + CFZ, particularly after 72 h. Thus, these results demonstrated that the chitosan derivatives CS-AC, CS-AC-EN, and CS-AC-DIEN are promising materials for the controlled release of the CFZ drug in both gastric and intestinal environments. Finally, when combined with CFZ, these derivatives exhibit significant potential for antibacterial applications against Gram-positive and Gram-negative bacteria.
